# Genetic diversity and population genetics of large lungworms (*Dictyocaulus*, Nematoda) in wild deer in Hungary

**DOI:** 10.1007/s00436-016-5088-0

**Published:** 2016-05-06

**Authors:** Zoltán Ács, Alexander Hayward, László Sugár

**Affiliations:** 1 Department of Wildlife Biology and Ethology, Faculty of Animal and Environmental Sciences, Kaposvar University, 7400 Kaposvár, Hungary; 2 Centre for Ecology and Conservation, University of Exeter, Cornwall Campus, Penryn, TR10 9E2 UK

**Keywords:** *Dictyocaulosis*, Helminth, Lungworm, Deer, Population genetics

## Abstract

*Dictyocaulus* nematode worms live as parasites in the lower airways of ungulates and can cause significant disease in both wild and farmed hosts. This study represents the first population genetic analysis of large lungworms in wildlife. Specifically, we quantify genetic variation in *Dictyocaulus* lungworms from wild deer (red deer, fallow deer and roe deer) in Hungary, based on mitochondrial *cytochrome c oxidase subunit 1* (*cox1*) sequence data, using population genetic and phylogenetic analyses. The studied *Dictyocaulus* taxa display considerable genetic diversity. At least one cryptic species and a new parasite–host relationship are revealed by our molecular study. Population genetic analyses for *Dictyocaulus eckerti* revealed high gene flow amongst weakly structured spatial populations that utilise the three host deer species considered here. Our results suggest that *D. eckerti* is a widespread generalist parasite in ungulates, with a diverse genetic backround and high evolutionary potential. In contrast, evidence of cryptic genetic structure at regional geographic scales was observed for *Dictyocaulus capreolus*, which infects just one host species, suggesting it is a specialist within the studied area. *D. capreolus* displayed lower genetic diversity overall, with only moderate gene flow compared to the closely related *D. eckerti*. We suggest that the differing vagility and dispersal behaviour of hosts are important contributing factors to the population structure of lungworms, and possibly other nematode parasites with single-host life cycles. Our findings are of relevance for the management of lungworms in deer farms and wild deer populations.

## Introduction

*Dictyocaulus* lungworms live as parasites in the lower airways of ruminants. Worm burden (worm abundance per individual host) varies from mild to heavy and can result in severe host pathology, a condition referred to as ʽdictyocaulosis’. For example, *Dictyocaulus viviparus* (Bloch, 1782) causes severe and frequently fatal bronchitis and pneumonia in cattle (termed ʽhusk’), which is of serious veterinary and agricultural importance due to animal welfare issues, reduced production yields and costs associated with treatment (David 1997; Ploeger 2002; Kutzer 1988; Wooley 1997). *Dictyocaulus eckerti* Skrjabin, 1931 is the major parasite of importance in farmed deer, and heavy infestations in young hosts can lead to anaemia and death, leading to substantial negative consequences for the farming industry (Mason 1994; Sugár 1997). *Dictyocaulus* species are also believed to result in parasitic bronchitis in a wide variety of wild ruminants (Urquhart et al. 1996).

*Dictyocaulus* species are classified into the monogeneric Dictyocaulidae family and the Trichostrongyloidea superfamily (but see Höglund et al. 2003; Chilton et al. 2006) and have a direct life cycle (Kassai 1999). The genus *Dictyocaulus* contains seven species: *Dictyocaulus africanus* (Gibbons & Khalil, 1988), *Dictyocaulus arnfieldi* (Cobbold, 1884), *Dictyocaulus cameli* (Boev, 1951), *Dictyocaulus capreolus* (Gibbons & Höglund, 2002), *D. eckerti*, *Dictyocaulus filaria* (Rudolphi, 1809) and *D. viviparus* (Durette-Desset et al. 1988; Gibbons and Khalil 1988; Gibbons and Höglund 2002). In order to confidently separate amongst *Dictyocaulus* species, molecular methods are necessary due to difficulties associated with morphological identification (Divina et al. 2000; Höglund et al. 2003). The commonly applied molecular techniques for this purpose are amplification of a specific gene fragment, followed by restriction enzyme digestion or single nucleotide polymorphism analysis, as well as polymerase chain reaction (PCR) assays of *18S*, *28S* and *ITS* ribosomal DNA (rDNA) sequences (Schnieder et al. 1996; Epe et al. 1997; Höglund et al. 1999, 2008; Johnson et al. 2004; Carreno et al. 2009).

Conventional chemotherapeutic treatments to reduce *Dictyocaulus* infections are costly and must be repeated frequently. Consequently, there is a current research focus on developing vaccines to control *Dictyocaulus* lungworms (McKeand 2000; Strube et al. 2015). Thus, knowledge of genetic variation and the population genetic structure in *Dictyocaulus* lungworms is important if we are to develop effective measures of control. Evidence from studies of diverse parasitic nematodes suggest several patterns of population structure can occur in such species (Blouin et al. 1995, 1999; Gilabert and Wasmuth 2013). Generally, the population structure in parasitic nematodes, in terms of genetic diversity and divergence, is influenced by levels of gene flow, and so an important factor is often host mobility (Blouin et al. 1995, 1999; Hawdon et al. 2001; Braisher et al. 2004).

Currently, population genetic studies of *Dictyocaulus* nematodes have been restricted to the cattle lungworm (*D. viviparus*) amongst Swedish farms (Hu et al. 2002; Höglund et al. 2004, 2006, 2008). Cattle lungworms display low levels of gene flow and high population genetic structure compared to other worms in the highly diverse trichostrongylid family, but similar levels to the less diverse, highly structured nematode parasite populations known from plants and insects (Hugall et al. 1994; Blouin et al. 1999; Höglund et al. 2004). The majority of trichostrongilid parasite populations appear to have high genetic diversity and little genetic structuring, suggestive of panmictic populations (Blouin et al. 1995, 1998; Archie and Ezenwa 2011).

Mitochondrial DNA sequences are considered particularly useful for studying interspecific and intraspecific variation because of their high evolutionary rates, predominantly maternal inheritance and limited recombination (Blouin 1998, 2002; Zhu et al. 2000). Variation in the nematode mitochondrial genome appears to be somewhat higher than in many other animal groups (Blouin 1998). Therefore, mitochondrial markers are a suitable and commonly applied choice to conduct estimates of population genetic structure, recent phylogeny and gene flow amongst populations. Furthermore, mitochondrial protein coding genes have higher variation than ribosomal genes for *Dictyocaulus* specifically (Höglund et al. 2006). The mitochondrial *cytochrome c oxidase 1* locus *(cox1*) in particular is frequently used in population genetic studies because it exhibits a relatively high mutation rate, conserved primers are available, and the large amount of data available for other species provides a comparison of genetic variation and population structure. Indeed, the mitochondrial *cox1* gene has been employed in a variety of studies on parasite nematodes (Hawdon et al. 2001; Blouin 2002; Hu et al. 2002; Miranda et al. 2008).

In the present study, we analyse genetic diversity at *cox1* for *Dictyocaulus* lungworms parasitizing wild deer species in Hungary. It is important to extend research in this field so that general biological insights regarding the evolution and ecology of *Dictyocaulus* lungworms can be made (Höglund et al. 2003). Our study is the first attempt to analyse the population genetic structure of large lungworms living in wild hosts, and our specific objectives were to: (1) examine broad-scale evolutionary patterns amongst *Dictyocaulus* species in wild deer; (2) assess host relationships amongst the observed species; (3) identify genetic diversity, differentiation, geneflow and demographic history for recovered *Dictyocaulus* species; and (4) determine whether patterns are similar to those observed for *D. viviparus* in farmed cattle, which represent the only other *Dictyocaulus* species for which population genetic data are available.

## Materials and methods

### Sampling of parasites

**Fig. 1:**
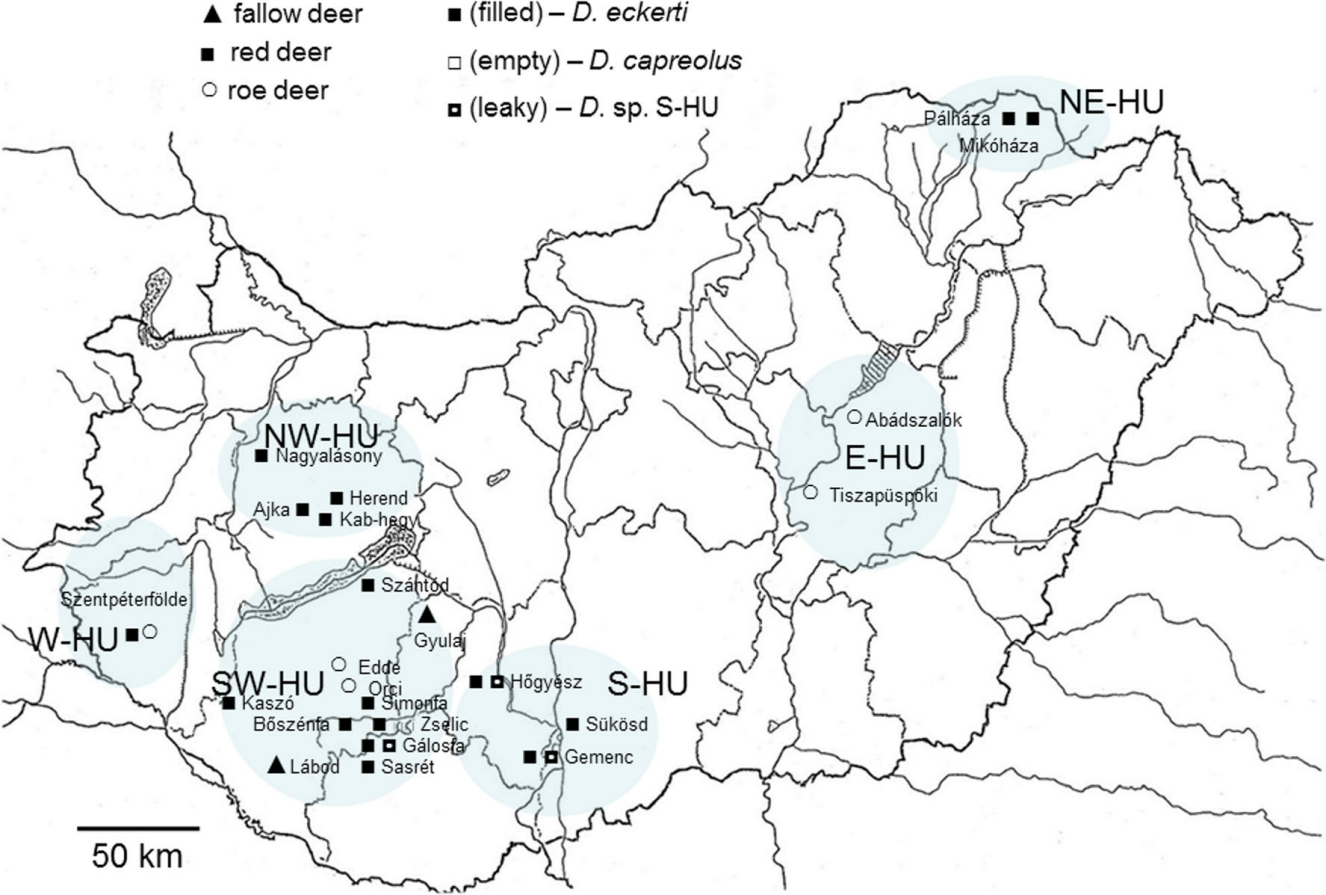
Map of collecting sites of *Dictyocaulus* in Hungary. Host species are indicated using different symbols (*triangle*: fallow deer; *square*: red deer; *circle*: roe deer), as are lungworm species (*filled symbol*: *D. eckerti*; *empty symbol*: *D. capreolus*; *leaky symbol*: *D.* sp. S-HU)

Adult lungworms were collected from the trachea and bronchi of the following deer species harvested during hunting: fallow deer (*Dama dama*), red deer (*Cervus elaphus*) and roe deer (*Capreolus capreolus*). Samples were taken from 23 sites in Hungary and one locality in neighbouring Romania (Fig. 1). Collecting sites were separated by distances ranging from 20 to 415 km in Hungary. The locality in Romania was situated in the Eastern Carpathians at Kászon, at a distance from the most eastern locality in Hungary (Mikóháza) of 460 km. Worms were collected during the period 2004–2015. After collection, individual worms were washed with physiological saline to avoid contamination and preserved in absolute alcohol at −20 °C. Specimens were randomly selected for subsequent genetic analyses, and a portion of approximately 1 cm was excised from the midbody of each individual for DNA extraction. Consequently, the anterior head and posterior end remained intact for morphological examination. Lungworms were identified to the genus level using taxonomic keys (Divina et al. 2000; Gibbons and Khalil 1988; Gibbons and Höglund 2002). To identify dictyocaulids to the species level, DNA sequencing of the internal transcribed spacer 2 (*ITS2*) of the nuclear ribisomal DNA was conducted (following Johnson et al. 2004) for selected samples from each clade (sample IDs: D18, D24 and D82) and compared to sequences of currently known lungworm species using a nucleotide BLAST search in Genbank (https://blast.ncbi.nlm.nih.gov).

### DNA sequences

Total genomic DNA was extracted from each worm specimen using a spin-column-based extraction method (DNeasy Tissue Kit, Qiagen, Germany) following the manufacturer’s protocol. Each DNA sample was eluted using 200 μl of EA buffer, as supplied in the kit, and subsequently stored at −20 °C. The *cytochrome c oxidase subunit 1* (*cox1*) gene fragment was amplified using the universal barcoding primers LCO1490 5′-GGTCAACAAATCATAAAGATATTGG-3′ and HCO2198 5′-TAAACTTCAGGGTGACCAAAAAATCA-3′ (Folmer et al. 1994). PCR conditions were as follows: each 25-μl reaction mixture contained 0.5 μl of each primer (10 μM), 2 μl of dNTPs (2 mM), 2.5 μl of 10× PCR buffer, 0.8–2 μl of MgCl_2_ (1.5 mM), 1 U of Taq polymerase (Fermentas) and varying concentrations of DNA and dH_2_O depending on the quality of the DNA extraction. Samples lacking genomic DNA were included in each PCR amplification as negative controls, and no products were detected in these negative controls. Amplification was preceded by one cycle of initial denaturation at 94 °C for 120 s, followed by six cycles of 94 °C for 30 s, 50 °C for 90 s and 72 °C for 60 s, and then 36 cycles of 94 °C for 30 s, 55 °C for 90 s and 72 °C for 60 s, with a terminal extension of 72 °C for 5 min. The yield and quantity of DNA were analysed using ethidium bromide staining and agarose gel electrophoresis. PCR products were cleaned using shrimp alkaline phosphatase and *Eschericia coli* exonuclease I (Fermentas) and sequenced directly on an ABI Prism 3730 Genetic Analyser machine using ABI BigDye Terminator Sequencing chemistry. Purified PCR products were sequenced using the same primers as for the PCR reaction, in both directions to minimise PCR artefacts, ambiguities and base-calling errors. Chromatogram output was checked by eye using Bioedit v.7 (Hall 1999). In a small proportion of cases, direct sequencing of *cox1* PCR products revealed multiple fragments, suggesting contamination by host (deer) DNA. In such cases, these results were eliminated from the study. Since *cox1* is a protein coding gene, only specimens for which a single open reading frame (ORF) was identified were included in the analyses. In total, our analyses include 103 new sequences, each derived from a single worm specimen, as well as nine sequences retrieved from GenBank: accession nos. JX519460, KM359418, KM359416, KM359417 for *D. viviparus*; JX519459 for *D. eckerti* (cf. red deer); JX519458 for *Aelurostrongylus abstrusus*; GQ888714 for *Metastrongylus pudendotectus*; GQ888715 for *Metastrongylus salmi*; and KF481953 for *Protostrongylus rufescens*. The last four species are related to Dictyocaulidae within the Strongylida order, and their sequences were included as outgroups in the phylogenetic analysis. All sequences generated in this study were deposited in GenBank under accession nos. KT372244–KT372346 (*cox1*) and KT438069–KT438071 (*ITS2*; Table 1).

**Table 1:** List of sampling details of *Dictyocaulus* lungworms

Species of parasite	Collecting data of parasite (lungworm)					Data of host (deer)				GenBank accession no. of parasite *cox1* (ITS2)
	No. p	Locality	Region, county	Date	Sex of parasite	No. h	Species of host	Sex	Age	
*D. capreolus*	D22	Zselic	SW-HU, Somogy	2005.03.29	♀	G14	*C. elaphus*	–	Juvenile	KT372264
	D30	Szentpéterfölde	W-HU, Vas	2009.02.13	♂	G19	*C. capreolus*	♀	Juvenile	KT372266
	D33	Edde	SW-HU, Somogy	2010.02.09	♀	G22	*C. capreolus*	♀	Adult	KT372262
	D37	Edde	SW-HU, Somogy	2008.04.27	–	G26	*C. capreolus*	♂	Adult	KT372255
	D82	Szentpéterfölde	W-HU, Vas	2009.02.13	♀	G19	*C. capreolus*	♀	Adult	KT372267 (KT438071)
	D83	Szentpéterfölde	W-HU, Vas	2009.02.13	♀	G19	*C. capreolus*	♀	Adult	KT372256
	D93	Abádszalók	E-HU, J.N.Szolnok	2010.12.12	–	G50	*C. capreolus*	–	Juvenile	KT372257
	D94	Abádszalók	E-HU, J.N.Szolnok	2010.12.12	–	G50	*C. capreolus*	–	Juvenile	KT372261
	D96	Abádszalók	E-HU, J.N.Szolnok	2010.12.12	–	G50	*C. capreolus*	–	Juvenile	KT372251
	D104	Tiszapüspöki	E-HU, J.N.Szolnok	2010.12.23	♀	G51	*C. capreolus*	♂	Adult	KT372253
	D187	Orci	SW-HU, Somogy	2011.04.25	♀	G53	*C. capreolus*	♀	Juvenile	KT372265
	D188	Orci	SW-HU, Somogy	2011.04.25	♀	G53	*C. capreolus*	♀	Juvenile	KT372263
	D189	Abádszalók	E-HU, J.N.Szolnok	2011.04.25	♀	G54	*C. capreolus*	♂	Adult	KT372260
	D190	Abádszalók	E-HU, J.N.Szolnok	2011.04.25	♀	G54	*C. capreolus*	♂	Adult	KT372254
	D193	Abádszalók	E-HU, J.N.Szolnok	2011.04.25	♀	G55	*C. capreolus*	♂	Adult	KT372258
	D197	Abádszalók	E-HU, J.N.Szolnok	2011.04.25	♂	G56	*C. capreolus*	♂	Adult	KT372259
	D198	Abádszalók	E-HU, J.N.Szolnok	2011.04.25	♂	G57	*C. capreolus*	♂	Adult	KT372252
*D. eckerti*	D1	Herend	NW-HU, Veszprém	2005.12.10	♀	G1	*C. elaphus*	–	Adult	KT372337
	D2	Herend	NW-HU, Veszprém	2005.12.10	♀	G1	*C. elaphus*	–	Adult	KT372336
	D4	Herend	NW-HU, Veszprém	2005.12.10	♀	G2	*C. elaphus*	–	Adult	KT372331
	D5	Gyulaj	SW-HU, Somogy	2004.01.15	–	G3	*D. dama*	–	Adult	KT372328
	D11	Kab-hegy	NW-HU, Veszprém	2005.01.27	–	G6	*C. elaphus*	♂	Adult	KT372273
	D13	Lábod	SW-HU, Somogy	2005.03.09	♂	G7	*D. dama*	♀	Adult	KT372302
	D14	Lábod	SW-HU, Somogy	2005.03.09	♂	G7	*D. dama*	♀	Adult	KT372346
	D15	Lábod	SW-HU, Somogy	2005.03.04	♀	G8	*D. dama*	–	Adult	KT372320
	D19	Hőgyész	S-HU, Tolna	2006.01.14	♀	G11	*C. elaphus*	♂	Adult	KT372293
	D21	Sükösd	S-HU, Tolna	2006.01.12	♀	G13	*C. elaphus*	♀	Adult	KT372341
	D23	Zselic	SW-HU, Somogy	2005.03.29	♂	G14	*C. elaphus*	–	Juvenile	KT372311
	D24	Kászon	RO, E-Carpathians	2005.09.28	♀	G15	*C. capreolus*	♂	Adult	KT372268 (KT438070)
	D25	Kászon	RO, E-Carpathians	2005.09.28	–	G15	*C. capreolus*	♂	Adult	KT372314
	D26	Kászon	RO, E-Carpathians	2005.09.28	♀	G15	*C. capreolus*	♂	Adult	KT372287
	D27	Gálosfa	SW-HU, Somogy	2006.11.08	♀	G16	*C. elaphus*	–	Juvenile	KT372307
	D28	Gálosfa	SW-HU, Somogy	2006.11.08	♀	G17	*C. elaphus*	♀	Adult	KT372313
	D31	Gálosfa	SW-HU, Somogy	2007.12.07	♀	G20	*C. elaphus*	–	Juvenile	KT372319
	D34	Lábod	SW-HU, Somogy	2008.06.06	♀	G23	*C. elaphus*	–	–	KT372308
	D36	Bőszénfa	SW-HU, Somogy	2006.11.20	♀	G25	*C. elaphus*	♂	Adult	KT372277
	D41	Gemenc	S-HU, Tolna	2006.01.25	♀	G30	*C. elaphus*	♂	Adult	KT372292
	D43	Hőgyész	S-HU, Tolna	2006.01.12	–	G32	*C. elaphus*	♀	Adult	KT372343
	D45	Kab-hegy	NW-HU, Veszprém	2005.01.27	♀	G6	*C. elaphus*	♂	Adult	KT372272
	D47	Hőgyész	S-HU, Tolna	2006.01.14	♀	G11	*C. elaphus*	♂	Adult	KT372269
	D48	Hőgyész	S-HU, Tolna	2006.01.14	♂	G11	*C. elaphus*	♂	Adult	KT372291
	D49	Hőgyész	S-HU, Tolna	2006.01.14	♀	G11	*C. elaphus*	♂	Adult	KT372316
	D50	Hőgyész	S-HU, Tolna	2006.01.14	♀	G11	*C. elaphus*	♂	Adult	KT372317
	D52	Hőgyész	S-HU, Tolna	2006.01.14	–	G11	*C. elaphus*	♂	Adult	KT372315
	D53	Hőgyész	S-HU, Tolna	2006.01.14	♀	G11	*C. elaphus*	♂	Adult	KT372297
	D54	Hőgyész	S-HU, Tolna	2006.01.14	♀	G11	*C. elaphus*	♂	Adult	KT372295
	D55	Gemenc	S-HU, Tolna	2006.01.25	♀	G34	*C. elaphus*	♀	Adult	KT372279
	D56	Sükösd	S-HU, Tolna	2006.01.12	♂	G35	*C. elaphus*	♀	Adult	KT372305
	D57	Zselic	SW-HU, Somogy	2005.03.29	♀	G36	*C. elaphus*	–	Juvenile	KT372288
	D58	Zselic	SW-HU, Somogy	2005.03.29	♀	G14	*C. elaphus*	–	Juvenile	KT372339
	D59	Zselic	SW-HU, Somogy	2005.03.29	♀	G14	*C. elaphus*	–	Juvenile	KT372323
	D60	Zselic	SW-HU, Somogy	2005.03.29	♂	G14	*C. elaphus*	–	Juvenile	KT372294
	D62	Zselic	SW-HU, Somogy	2005.06.22	–	G37	*C. elaphus*	♀	Adult	KT372340
	D63	Zselic	SW-HU, Somogy	2005.06.22	♀	G37	*C. elaphus*	♀	Adult	KT372274
	D64	Zselic	SW-HU, Somogy	2005.04.20	♀	G38	*C. elaphus*	–	Juvenile	KT372345
	D66	Zselic	SW-HU, Somogy	2005.04.20	♀	G38	*C. elaphus*	–	Juvenile	KT372300
	D67	Kaszó	SW-HU, Somogy	2009.12.13	♀	G39	*C. elaphus*	♂	Juvenile	KT372329
	D68	Kaszó	SW-HU, Somogy	2009.12.13	♀	G39	*C. elaphus*	♂	Juvenile	KT372330
	D69	Kaszó	SW-HU, Somogy	2010.01.29	♀	G40	*C. elaphus*	–	Adult	KT372296
	D70	Kaszó	SW-HU, Somogy	2010.01.29	♀	G40	*C. elaphus*	–	Adult	KT372335
	D71	Kaszó	SW-HU, Somogy	2010.01.29		G41	*C. elaphus*	–	Adult	KT372310
	D79	Kab-hegy	NW-HU, Veszprém	2009.12.10	♀	G45	*C. elaphus*	♂	Adult	KT372322
	D80	Kab-hegy	NW-HU, Veszprém	2009.12.10	♀	G45	*C. elaphus*	♂	Adult	KT372344
	D81	Kab-hegy	NW-HU, Veszprém	2009.12.10	♀	G45	*C. elaphus*	♂	Adult	KT372342
	D113	Gemenc	S-HU, Tolna	2006.01.25	♀	G30	*C. elaphus*	♂	Adult	KT372324
	D121	Hőgyész	S-HU, Tolna	2006.01.12	–	G11	*C. elaphus*	♂	Adult	KT372325
	D133	Zselic	SW-HU, Somogy	2005.04.20	♀	G38	*C. elaphus*	–	Juvenile	KT372271
	D137	Zselic	SW-HU, Somogy	2005.04.20	♀	G38	*C. elaphus*	–	Juvenile	KT372270
	D140	Kászon	RO, E-Carpathians	2005.09.28	♂	G15	*C. capreolus*	♂	Adult	KT372321
	D146	Kászon	RO, E-Carpathians	2005.09.28	♀	G15	*C. capreolus*	♂	Adult	KT372281
	D150	Hőgyész	S-HU, Tolna	2006.01.12	♂	G11	*C. elaphus*	♂	Adult	KT372290
	D154	Hőgyész	S-HU, Tolna	2006.01.12	♀	G11	*C. elaphus*	♂	Adult	KT372283
	D155	Zselic	SW-HU, Somogy	2005.04.20	♀	G38	*C. elaphus*	–	Juvenile	KT372338
	D159	Gálosfa	SW-HU, Somogy	2007.12.07	♂	G20	*C. elaphus*	–	Juvenile	KT372278
	D169	Gálosfa	SW-HU, Somogy	2007.12.07	♂	G20	*C. elaphus*	–	Juvenile	KT372327
	D174	Lábod	SW-HU, Somogy	2005.03.04	♀	G8	*D. dama*	–	Adult	KT372282
	D175	Lábod	SW-HU, Somogy	2005.03.04	♀	G8	*D. dama*	–	Adult	KT372326
	D203	Ajka	NW-HU, Veszprém	2012.01.14	♀	G60	*C. elaphus*	–	Adult	KT372275
	D204	Ajka	NW-HU, Veszprém	2012.01.14	–	G60	*C. elaphus*	–	Adult	KT372286
	D257	Nagyalásony	NW-HU, Veszprém	2012.11.26	♀	G69	*C. elaphus*	♀	Juvenile	KT372280
	D260	Nagyalásony	NW-HU, Veszprém	2012.11.26	–	G69	*C. elaphus*	♀	Juvenile	KT372318
	D263	Lábod	SW-HU, Somogy	2013.01.20	♀	G71	*C. elaphus*	♂	Juvenile	KT372285
	D264	Lábod	SW-HU, Somogy	2013.01.20	♂	G71	*C. elaphus*	♂	Juvenile	KT372306
	D265	Lábod	SW-HU, Somogy	2013.01.20	♂	G71	*C. elaphus*	♂	Juvenile	KT372309
	D268	Lábod	SW-HU, Somogy	2013.01.20	♀	G72	*C. elaphus*	♀	Juvenile	KT372284
	D270	Lábod	SW-HU, Somogy	2013.01.20	–	G73	*C. elaphus*	♀	Adult	KT372303
	D271	Lábod	SW-HU, Somogy	2013.01.20	–	G73	*C. elaphus*	♀	Adult	KT372304
	D272	Simonfa	SW-HU, Somogy	2013.01.25	♀	G74	*C. elaphus*	♀	Adult	KT372301
	D273	Simonfa	SW-HU, Somogy	2013.01.25	♀	G74	*C. elaphus*	♀	Adult	KT372289
	D274	Simonfa	SW-HU, Somogy	2013.01.25	♀	G74	*C. elaphus*	♀	Adult	KT372312
	D302	Pálháza	NE-HU, Zemplén	2014.09.23	♀	G83	*C. elaphus*	♂	Adult	KT372298
	D306	Pálháza	NE-HU, Zemplén	2014.09.23	♀	G83	*C. elaphus*	♂	Adult	KT372299
	D307	Mikóháza	NE-HU, Zemplén	2014.09.24	♀	G84	*C. elaphus*	♂	Adult	KT372333
	D308	Mikóháza	NE-HU, Zemplén	2014.09.24	♀	G84	*C. elaphus*	♂	Adult	KT372276
	D309	Mikóháza	NE-HU, Zemplén	2014.09.24	♀	G84	*C. elaphus*	♂	Adult	KT372332
	D311	Mikóháza	NE-HU, Zemplén	2014.09.24	♀	G84	*C. elaphus*	♂	Adult	KT372334
*D*. sp. S-HU	D18	Hőgyész	S-HU, Tolna	2006.01.12	–	G10	*C. elaphus*	♂	Adult	KT372247 (KT438069)
	D42	Gemenc	S-HU, Tolna	2006.01.10	♀	G31	*C. elaphus*	♀	Adult	KT372244
	D44	Hőgyész	S-HU, Tolna	2006.01.12	♀	G33	*C. elaphus*	♂	Adult	KT372249
	D46	Hőgyész	S-HU, Tolna	2006.01.12	♀	G32	*C. elaphus*	♀	Adult	KT372245
	D118	Gemenc	S-HU, Tolna	2006.01.10	♀	G31	*C. elaphus*	♀	Adult	KT372250
	D119	Gemenc	S-HU, Tolna	2006.01.10	♀	G31	*C. elaphus*	♀	Adult	KT372246
	D166	Gálosfa	SW-HU, Somogy	2007.12.07	♀	G20	*C. elaphus*	–	Juvenile	KT372248

: hermaphrodite, juvenile: less than 1 year old, adult: more than 1 year old

*No. p* identification number of parasite individual, *No. h* identification number of host individual

### Evolutionary relationships

Sequences of *cox1* were aligned using ClustalX version 2.0 (Thompson et al. 1997). To infer the most likely model of sequence evolution for the *cox1* dataset, we used the Akaike and Bayesian information criteria (AIC and BIC) as implemented in MODELTEST (Posada and Crandall 1998) and MEGA6 (Tamura et al. 2013). The best-fitting model of sequence evolution was the Tamura–Nei model with gamma-distributed rate variation and a proportion of invariable sites (TN93+G+I) according to both AIC and BIC. Mitochondrial sequences evolve relatively rapidly (in comparison to many nuclear genes), and this can affect the signal-to-noise ratio for phylogenetic datasets, which in severe cases can lead to the inference of erroneous relationships amongst taxa. To investigate this possibility, we implemented a test of mutational saturation in the DAMBE5 (Xia 2013) across each codon position for our dataset. To examine the evolutionary relationships amongst lungworm samples, we reconstructed a maximum likelihood phylogenetic tree using MEGA. Bootstrap clade support was inferred using 1000 bootstrap replicates.

### Population genetic analysis

To infer the population structure of lungworms and examine the processes that have shaped present distributions, several analyses of amplified *cox1* sequences were performed. Genetic diversity values, including polymorphic sites (*S*), *GC* nucleotide content, haplotype number (*H*), haplotype diversity (*H*_d_), average number of nucleotid differences within groups (*K*) and nucleotide diversity (π), were calculated within species and populations using DnaSP version 5 (Librado and Rozas 2009). All estimates were calculated using DnaSP, including those described below.

We measured genetic variation at four levels (individual host, host species, locality and region) relative to the entire population (for group specification, see Table 2), as well as making between-species comparisons. Population structure and gene flow were evaluated by analysis of molecular variance. Genetic differentiation between populations of each lungworm species was estimated using *F*_ST_ (Hudson et al. 1992). Nei’s *G*_ST_ was calculated to estimate population differentiation based on differences in allele frequencies (Nei 1973). Estimates of population differentation were based on nucleotide diversity using *N*_ST_ (Lynch and Crease 1990). Additionally, we also calculated *N*_*m*_, which is the mean per generation estimate of the absolute number of migrants exchanged amongst populations as inferred from *F*_ST_. These analyses test whether the a priori populations defined by collecting locality, region and host represent distinct genetic groups.

**Table 2:** Genetic diversity of *Dictyocaulus* populations from wild deer based on *cox1* DNA sequences

Parasite species	Populations defined by	Population		*H*/*N*	*S*	*H*_d_	*K*	π
*D. eckerti*	Host individual	G8		2/3	14	0.6667	9.33	0.0142
		G11		7/11	29	0.8909	11.13	0.0169
		G14		4/4	18	1	10.00	0.0152
		G15		5/5	34	1	15.7	0.0239
		G20		2/3	16	0.6667	10.67	0.0162
		G38		5/5	24	1	12.4	0.0189
		G45		3/3	11	1	7.33	0.0112
		G71		3/3	15	1	10.00	0.0152
		G74		3/3	20	1	13.33	0.0203
		G84		2/4	13	0.50	6.5	0.0099
	Locality	Herend	NW-HU	3/3	8	1	5.33	0.0081
		Ajka–Kab-hegy		6/7	37	0.9524	14.57	0.0222
		Nagyalásony		2/2	14	1	14.00	0.0213
		Lábod	SW-HU	8/12	30	0.9240	10.17	0.0155
		Zselic–Gálosfa		15/17	39	0.9779	12.06	0.0183
		Bőszénfa–Simonfa		4/4	21	1	11.83	0.0183
		Kaszó		4/5	21	0.9	10.20	0.0155
		Hőgyész	S-HU	8/12	29	0.9091	11.38	0.0173
		Gemenc–Sükösd		5/5	26	1	11.80	0.0180
		Pálháza–Mikóháza	NE-HU	3/6	18	0.73	7.80	0.0119
		Kászon	RO	5/5	34	1	15.70	0.0239
	Host species	Red deer		44/68	87	0.977	12.10	0.0184
		Falow deer		5/6	24	0.933	9.93	0.0151
		Roe deer		5/5	34	1	15.7	0.0239
	Region	NW-HU		10/12	41	0.9697	12.17	0.0185
		SW-HU		28/38	58	0.9744	11.64	0.0181
		S-HU		12/18	39	0.9346	11.88	0.0181
		NE-HU		3/6	18	0.7333	7.80	0.0119
		RO, E-Carpathians		5/5	34	1	15.70	0.0239
*D. capreolus*	Host individual	G19 (W-HU)		2/3	4	0.6667	2.67	0.0041
		G50 (E-HU)	3/3	15	1	10.00	0.0152	
	Locality	Szentpéterfölde	W-HU	2/3	4	0.6667	2.67	0.0041
		Abádszalók	E-HU	8/8	23	1	8.21	0.0125
		Edde	SW-HU	2/2	3	1	3.00	0.0046
		Orci		1/2	0	1	0	0
	Region	W-HU		2/3	4	0.6667	2.67	0.0041
		E-HU		8/9	23	0.9722	7.83	0.0119
		SW-HU		3/5	4	0.7	1.8	0.0027
*D*. sp. S-HU	Locality	Hőgyész		3/3	11	1	7.33	0.0112
		Gemenc		3/3	13	1	8.67	0.0132
		Gálosfa		1	–	–	–	–

Populations of the three lungworm species were defined by individual host animal, collecting locality, collecting region and host species

*S* number of variable sites, *N* number of sequences obtained, *H* number of haplotypes, *H*_d_ haplotype diversity, *K* average number of nucleotide diferences, π nucleotide diversity, *E* East Hungary, *NW* northwest Hungary, *S* South Hungary, *SW* southwest Hungary, *NE* northeast Hungary, *W* West Hungary, *RO* Romania

The population history of *Dictyocaulus* species was also estimated. Tajima’s *D* (Tajima 1989) and Fu’s *F*_s_, which is based on the haplotype frequency distribution (Fu 1997), were used to identify genetic signals of deviation from neutrality and population decline or expansion. Tajima’s *D* is based on the difference between estimates of the number of segregating sites and the average number of pairwise differences. These values were estimated via 10,000 computer simulations based on observed pairwise differences. Positive values of both parameters indicate population decline, whilst negative values suggest population expansion. Fu’s *F*_s_ test is more sensitive to demographic changes (Ramos and Rozas 2002). Mismatch distribution analyses (examining the distribution of pairwise differences) are frequently used to estimate population history. Such analyses compare the frequency distribution of pairwise differences between haplotypes with that expected under a model of population expansion (Slatkin and Hudson 1991). The multimodal mismatch distribution predicts that the population has a stable size over its history. Sudden demographic expansion leads to a unimodal distribution of pairwise differences. The smoothness of the mismatch distribution was quantified by the raggedness statistic *r* (ranked pairwise differences in the population), as described by Harpending et al. (1993). The time (*t*) to the most recent common ancestor (tMRCA) for our samples was also estimated. This estimates the number of generations since the population expanded and was calculated from the peak distribution (*τ*) using the equation: *t* = *τ*/2*μ* (Li 1977). The parameter *μ* is the mutation rate per gene per generation and is obtained by multiplying the mutation rate per site per generation by the number of nucleotides in the studied fragment (657 bp in this case). The mutation rate of substitutions per site per generation was estimated using values for the mitochondrial DNA (mtDNA) of *Caenorhabditis elegans*: 1.57 × 10^−7^ ± 3.1 × 10^−8^ (Denver et al. 2000). Optimally, *D. viviparus* requires 3–4 weeks to develop from an egg to a mature adult (Kassai 1999; Johnson et al. 2004); however, environmental- and host-related factors can delay its life cycle by an additional 3–4 weeks. The reproductive season for dictyocaulid worms occurs during April–October in Hungary, leading to an estimate of four generations per year. The number of generations since population expansion (*t*) divided by generations per year gives an estimate of time in terms of number of years.

## Results

### Sequence analyses and evolutionary relationships

A total of 103 *cox1 Dictyocaulus* sequences were amplified. Each sequence originates from a single lungworm specimen. In total, lungworms were collected from 47 individual deer (Table 1). Collection localities are grouped according to region (Fig. 1). All *cox1* sequences were of the same length (657 bp) and could be aligned unambiguously. The resultant *cox1* alignment corresponds to positions 69–725 bp of the complete mitochondrial genome sequence of *D. eckerti* cf. red der (GenBank accession no. JX519459; Gasser et al. 2012). Based on the invertebrate mitochondrial genetic code, all amplified *cox1* sequences possessed a single ORF without the existence of stop codons. Nucleotide composition was heavily biased towards A and T bases, as is usual for nematode mtDNA (G+C content, 0.299–0.337; Table 3; Blouin et al. 1998). Tests of mutational saturation for the analysed *cox1* fragment, as well as each codon position individually, were negative (*P* < 0.0001).

**Table 3:** Genetic diversity of *Dictyocaulus* lungworms based on mitochondrial DNA sequences

Parasite	Gene	Examined sites (bp)	*S* (%)	*G*+*C* cont.	*H*/*N*	*H*_d_	*K*	π	*F*_s_	*D*	Reference
*D. eckerti*	*cox1*	657	92 (14)	0.337	51/79	0.974	12.08	0.0184	−23.85*	−1.31*	This study
*D. capreolus*	*cox1*	657	30 (4.6	0.299	13/17	0.963	5.662	0.0086	−4.48*	−1.47*	This study
*D*. sp. S-HU	*cox1*	657	19 (2.9)	0.302	6/7	0.952	6.810	0.0104	−0.53*	−0.93*	This study
*D. viviparus*	*cox1*	393	15 (3.8)	0.31	12/252	0.766	–	0.0058	–	–	Hu et al. (2002)
	*cox3*	375	17 (4.5)	0.265	7/72	0.73	–	0.013	5.66	1.12	Höglund et al. (2006)
	*nad5*	395	23 (5.8)	0.315	10/72	0.85	–	0.014	3.38	0.43	Höglund et al. (2006)
	*rrnL*	457	6 (1.3)	0.213	6/72	0.72	–	0.0039	1.58	0.54	Höglund et al. (2006)
	*trna*	309	6 (1.9)	0.179	8/72	0.82	–	0.0058	−0.29	0.60	Höglund et al. (2006)
	Combined *cox3*-*nad5*-*rrnL*-*trna*	1542	52 (3.4)	0.245	12/72	0.91	–	0.0090	10.9	0.79	Höglund et al. (2006)

*S* number of variable sites, *N* number of sequences obtained, *H* number of haplotypes, *H*_d_ haplotype diversity, *K* average number of nucleotide differences, π nucleotide diversity, *F*_s_ Fu’s *F*_s_ statistic (neutrality test), *D* Tajima’s *D* statistic

*P > 0.10 (not significant)

**Fig. 2:**
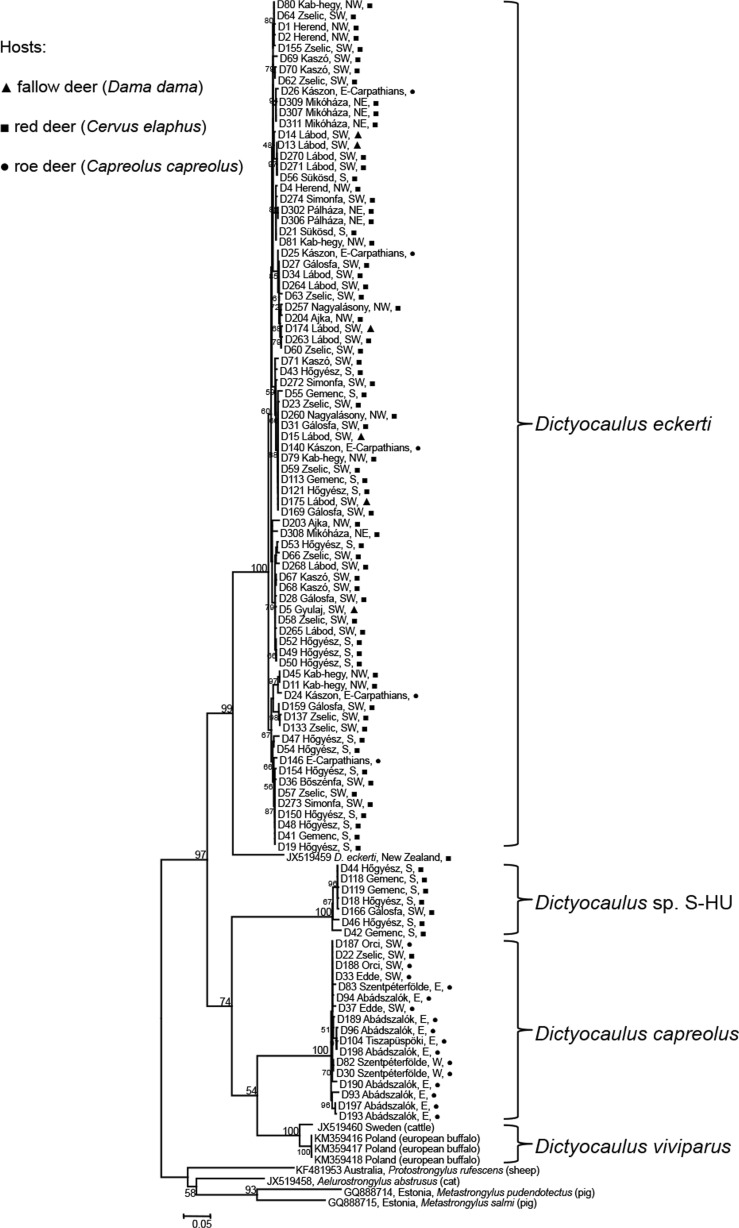
Maximum likelihood phylogenetic tree constructed using the mitochondrial *cox1* gene for 103 *Dictyocaulus* lungworms originating from Hungary and five lungworms from GenBank indicated by their *accession numbers* (one dictyocaulid worm of red deer in New Zealand and four sequences of *D. viviparus*). Lungworms were collected from hunted deer (fallow, red and roe deer), indicated by *triangle*, *square* and *circle*, respectively. Geographical collecting regions are indicated for each sample

Amplified *Dictyocaulus cox1* sequences grouped into four main clades according to maximum likelihood phylogenetic analysis, revealing that lungworms collected from wild deer belong to three distinct clades (Fig. 2). Sequence differences between clades were high (Table 4) compared to within-group variability (Table 3), suggesting the clades represent separate species: between-clade sequence differences exceeded 10 %, which is an empirical limit applied to species differentiation for nematodes (Blouin 2002). Additionally, *ITS2* sequences of selected samples from two clades (D24—KT438070 and D82—KT438071) showed high similarity to *D. eckerti* (96 and 100 % nucleotide identity; GenBank accession no. U37716; Epe et al. 1997) and *D. capreolus* (GenBank accession no. AF105255; Höglund et al. 1999), identifying the clades as *D. eckerti* and *D. capreolus*, respectively. An *ITS2* sequence for the D18 sample (KT438069) from the additional clade did not show close similarity to any currently known *Dictyocaulus* species; therefore, we consider it an unknown, probably undescribed species and refer to it here as *D*. sp. S-HU (reflecting the collecting region, South Hungary). Additionally, the lungworm sequence collected from a red deer in New Zealand (JX519459) is divergent with respect to the Hungarian *D. eckerti* samples, and the 0.094 mean pairwise sequence difference between the New Zealand sample and Hungarian sequences within the *D. eckerti* clade suggests these sequences may belong to different species.

**Table 4:** DNA divergence between lungworm species based on mitochondrial *cox1* sequences

Sites *Dxy*/*K*	*D. capreolus*	*D. eckerti*	*D.* sp. S-HU
*D. capreolus* (*n* = 17)		89.02	83.13
*D. eckerti* (*n* = 79)	0.1355		91.16
*D.* sp. S-HU (*n* = 7)	0.1265	0.1388	

The average number of nucleotide substitutions per site (*Dxy*) between species are shown in the lower left corner, whereas the average number of nucleotide differences (*K*) between species are displayed in the upper right corner

The structure of our *cox1* tree indicates high genetic differentiation between the three *Dictyocaulus* species from wild deer, but little or no differentiation within each species according to locality or geographical region (Fig. 2). *D. eckerti* was the most prevalent lungworm species and is represented by 79 sequences collected across 20 sampling localities from five geographical regions in Hungary (Fig. 1) and one locality in the Eastern Carpathians in Romania. Sequences of *D. eckerti* were recovered from all three deer species examined; however, no host structuring was apparent (Fig. 2). Additionally, specimens of *D. eckerti* were predominantly recovered from red deer: 32 red deer produced 68 worms, with only six worms recovered from three fallow deer and five worms from one roe deer. The 17 sequences obtained for *D. capreolus* were sampled from five localities and three geographical regions. Of the 17 *D. capreolus* worms included here, 16 originated from 10 roe deer, whilst only one worm was collected from red deer. The seven sequences of the *D*. sp. S-HU isolates were collected exclusively from red deer within three localities and two regions.

### Genetic diversity

Interspecific pairwise sequence distances between sequences from separate *Dictyocaulus* clades (12.6–13.8 %; Table 4) are one magnitude higher than intraspecific variation (0.8–1.8 %; Table 3), indicating substantial isolation amongst the three species examined. The levels of genetic diversity for *D. eckerti*, *D. capreolus* and *D*. sp. S-HU were determined using the statistics listed in Table 3 (*H*_d_, *K* and π). The mean nucleotide differences and nucleotide diversity for *D. eckerti* were approximately two times higher than those for *D. capreolus* and *D*. sp. S-HU (the mean nucleotide diversity, π, for *D. eckerti* was 0.018). The genetic diversity of *D. eckerti* within populations was consistent (range, 0.0081–0.0239) and much higher than for *D. capreolus* (the overall nucleotide diversity for *D. capreolus* was 0.0086 and that within populations ranged between 0.0027 and 0.0152). *H*_d_ values are close to 1 for all examined species, showing a diverse haplotype distribution. The number of haplotypes for *D. eckerti*, *D. capreolus* and *D*. sp. S-HU were 51, 13 and 6, respectively, and there were many polymorphic sites (2.9–14 %). Most haplotypes were represented by a single specimen (55 singletons, 79 % of lungworms from all three species). The most common haplotype of *D. eckerti* (HP5) comprises samples distributed through four regions (NW-HU, S-HU, SW-HU and RO).

### Genetic structure and gene flow across spatial distribution

Population genetic analyses were conducted for the *Dictyocaulus* species separately at several study levels. Because *D*. sp. S-HU is represented by relatively few samples, we focus our analyses on the datasets of *D. eckerti* and *D. capreolus*. The lungworm species examined showed different population genetic structures. The genetic structure of *D. eckerti* was low, with population estimates of *F*_ST_, *G*_ST_ and *N*_ST_ consistently within the range 0.034–0.050 at all levels (Table 5). Consequently, gene flow estimator (*N*_*m*_) values were high, indicating high gene flow amongst a priori defined populations. Estimated pairwise *F*_ST_ between populations of *D. eckerti* defined by collecting region ranged from 0.0037 to 0.0598 (Table 6). The highest genetic differences were found between N-HU and NW-HU regional populations. Interestingly, the geographically distant samples from the Eastern Carpathians did not correspond to the most isolated population and are incorporated within Hungarian populations (Fig. 2). *D. capreolus* showed moderate genetic structure at the regional scale. The *D. capreolus* dataset did not indicate substantial genetic structure at either the infrapopulation (host individual) or locality levels. Based on roughly equal pairwise *F*_ST_ calculations, the three *D. capreolus* populations are equally isolated from each other (Table 6). The overall population structure estimator values ranged between 0.133 and 0.153 (Table 5), and indications of moderate gene flow (*N*_*m*_ = 3.27) suggest that *D. capreolus* has intermediate genetic structure. The samples of *D*. sp. S-HU grouped into two populations, revealing limited genetic differences, but this result should be regarded cautiously due to the small sample size.

**Table 5:** Gene flow estimates for *Dictyocaulus* lungworms based on mtDNA sequences

Parasite	Populations defined by	Gene	*N*	No. of Populations	*H*	*F*_ST_	*G*_ST_	*N*_ST_	*N*_*m*_
*D. eckerti*	Region	*cox1*	79	5	51	0.0425	0.0346	0.0423	11.27
	Locality	*cox1*	78	11	50	0.0445	0.0385	0.0443	10.74
	Host species	*cox1*	79	3	51	−0.0273	0.0280	−0.0272	17.34
	Individual hosts	*cox1*	40	9	27	0.0500	0.0365	0.0501	13.20
*D. capreolus*	Region	*cox1*	17	3	13	0.1528	0.1328	0.1518	3.27
*D*. sp. S-HU	Region	*cox1*	6	2	5	−0.0909	−0.0588	−0.0900	−9.00
*D. viviparus* ^a^	Farms	*cox1*	252	17	12	0.77	0.7272	0.6589	–
*D. viviparus* ^b^	Farms	*cox3*	72	9	7	0.70	0.72	0.70	0.10
		*nad5*	72	9	10	0.73	0.71	0.73	0.09
		*rrnL*	72	9	6	0.80	0.80	0.80	0.06
		*trna*	72	9	8	0.74	0.69	0.74	0.09

Negative values result from unequal sample sizes

*N* number of sequences obtained, *H* number of haplotypes*, F*_ST_, *G*_ST_, *N*_ST_ fixation indices, *Nm* number of migrants per generation

^a^Data are from Hu et al. (2002)

^b^Data are from Höglund et al. (2006)

**Table 6:** Pairwise *F*_ST_ values between populations of *D. capreolus* and *D. eckerti* defined by collecting geographic region and host species

Species	Populations defined by	Pairwise comparison	*F*_ST_
*D. eckerti*	Region	NW-HU–S-HU	0.0584
		NW-HU–SW-HU	−0.0037
		NW-HU–Carpathians	−0.0284
		NW-HU–N-HU	−0.0598
		S-HU–SW-HU	0.0073
		S-HU–Carpathians	−0.0535
		S-HU–N-HU	0.0256
		SW-HU–Carpathians	0.0204
		SW-HU–N-HU	0.0048
		N-HU–Carpathians	−0.0336
	Host species	Red deer–fallow deer	0.0026
		Red deer–roe deer	0.0550
		Fallow deer–roe deer	0.0199
*D. capreolus*	Region	SW-HU–W-HU	0.1625
		SW-HU–E-HU	0.1500
		W-HU–E-HU	0.1512

### Gene flow across host species

Genetic structure was tested for *D. eckerti* samples collected from three host species (fallow deer, red deer and roe deer). Maximum likelihood phylogenetic analyses revealed that samples of *D. eckerti* lungworms grouped into subclusters, which were not correlated with the host species (Fig. 2). Haplotypes from different host species were randomly distributed across the *D. eckerti* clusters. In addition, there was no evidence for genetic structuring within host species based on *F*_ST_, *G*_ST_ or *N*_ST_ (Table 5). The low pairwise *F*_ST_ values between host-defined populations (Table 6) and the high rate of overall gene flow (*N*_*m*_ = 17.34) between host species suggest that *D. eckerti* uses multiple hosts and has well-connected populations in Hungary and with the Carpathian population.

### Population history

**Fig. 3:**
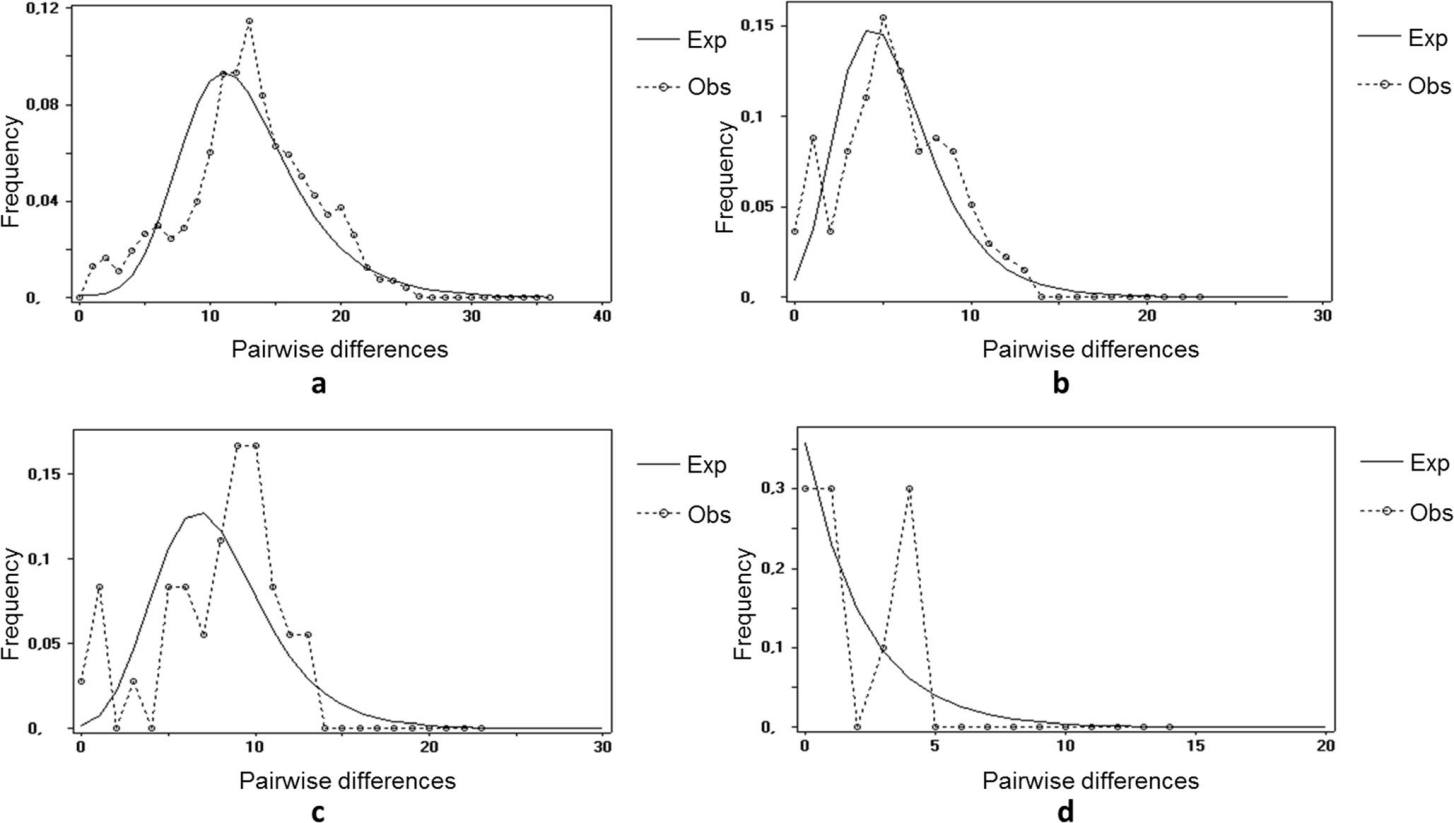
Observed and simulated (expected) mismatch frequency distributions under a model of population expansion for *D. eckerti* overall (**a**), *D. capreolus* overall (**b**) and the eastern population of *D. capreolus* (**c**) and under a model of constant population size for the western population of *D. capreolus* (**d**)

Tajima’s *D* neutrality tests showed negative values for all three *Dictyocaulus* species (weakly supported; Table 3). Similarly, Fu’s *F*_s_ test estimated negative values in all species overall, although none were significant. Neutrality tests for *D. eckerti* indicated strong departures from a mutation-drift equilibrium (Table 3). Deviations from equilibrium can stem from the effects of selection or demographic processes (population size change). The highest deviations from a mutation-drift equilibrium were recorded with Fu’s *F*_s_ test, which is one of the most sensitive tests for detecting demographic changes. Therefore, we assumed a demographic process was the most likely explanation for these results and proceeded to estimate the magnitude of historical population size change. Negative values for Tajima’s *D* and Fu’s *F*_s_ might suggest a population-wide demographic change or a recent range expansion for *D. capreolus* also. As expected under population expansion, the mismatch distributions for both species had an unimodal shape (Fig. 3a, b). The low raggedness value was also a sign of an expanded population. Also, *r* indices were low for both species (*r* = 0.0044 for *D. eckerti*; *r* = 0.0147 for *D. capreolus*).

Mismatch analyses were carried out for each species to date potential population expansion events. A tMRCA analysis was performed for all the samples in the case of *D. eckerti* since there was no or only weak genetic evidence for differentiated populations. The peak of the unimodal distribution (*τ*) was 9.529, corresponding to a population expansion approximately 46,000 generations ago, which equals ∼11,500 (9,600–14,400 CI) years ago, placing the event at the end of the last Ice Age, assuming four generations per year. These calculations assume that the *D. eckerti* population is at equilibrium. Based on the unimodal mismatch distribution, a similar analysis was performed for all samples of *D. capreolus* collectively, as well as separately for two populations. For all samples collectively, the estimate of *τ* was 3.669, suggesting an expansion estimate ∼4,500 years ago (3700–5500 CI). Because of evidence of moderate genetic structure for *D. capreolus* and the observation that eastern (E) and southwestern (SW) populations differ in genetic variability by about five times (Table 2), separate mismatch analyses were also carried out. The eastern population showed negative Tajima’s *D* and *F*_s_ values, −0.368 and −1.579, respectively (*P* > 0.10), and low raggedness (0.0363), indicating an expanded population. The unimodal mismatch distribution for the eastern population (Fig. 3c) also indicates an expanded population. According to the tMRCA analyses, the onset of the eastern population expansion was approximately 7000 (5900–8800 CI) years ago (*τ* = 5.827). However, the SW population of *D. capreolus* exhibited a low negative Tajima’s *D* (−0.4410, *P* > 0.10) and low positive Fu’s *F*_s_ (0.469, *P* > 0.10) with moderate raggedness (0.23), suggesting a roughly constant population size. The mismatch distribution of the SW population of *D. capreolus* (Fig. 3d) shows a multimodal distribution under a constant model, indicating occasional bottlenecks in population history.

## Discussion

In addition to their evolutionary and ecological relevance, information regarding the genetic variability of *Dictyocaulus* lungworms is of direct applied interest given their status as important parasites of farmed and wild animals. Amongst the macroparasites of deer (Cervidae), lungworms are believed to be pathogenic in farmed or fenced circumstances within temperate regions (Mason 1994; Sugár 1997). Prior to this study, population genetic analysis of dictyocaulid lungworms was restricted to the cattle lungworm, *D. viviparus*, in Sweden (Hu et al. 2002; Höglund et al. 2004, 2006, 2008). Studies on *D. viviparus* genetic diversity and gene flow amongst cattle farms have revealed a signature of strong population genetic structure, possibly influenced by human activities. Our survey aimed to reveal the population genetic structure of *Dictyocaulus* lungworms in natural wildlife, focussing on host deer species, at small and medium geographic scales.

Phylogenetic analyses reveal that *Dictyocaulus* sequences group into three strongly supported clades (100 % bootstrap support). Given the patterns of sequence divergence within (<2 %) and between clades (>13 %), our results strongly suggest that these three clades correspond to separate lungworm species in Hungary. Whilst support for our clades of interest is strong, the values amongst major clades are poor, limiting our ability to elucidate evolutionary relationships amongst them. A previous phylogenetic analysis of European *Dictyocaulus* species using rDNA *ITS2* recovered a different pattern from that observed in our results, with *D. capreolus* more basal, although support amongst clades was similarly low (Höglund et al. 2003). We identify an undescribed species in our analysis, referred to here as *D.* sp. S-HU. Interestingly, Höglund et al. (2003) also noted an undescribed species in their phylogenetic study of European *Dictyocaulus*. However, the undescribed species was recovered from fallow deer, whereas *D.* sp. S-HU was collected from red deer here. Thus, it is clear that there is currently at least one undescribed species of *Dictyocaulus* present in European deer. In the future, efforts should be made to compare these lineages, to examine whether they represent the same or different cryptic species, with formal descriptions following. Additionally, further sampling of New Zealand lungworms (and additional European sampling) would be of interest to determine the origin and identity of these worms.

Regarding host relationships, *D. eckerti* is a frequent parasite in wild and semi-domesticated hosts and is recorded from several host species: fallow deer, hog deer (*Axis porcinus*), Indian muntjac (*Muntiacus muntjak*), moose (*Alces alces*), musk ox (*Ovibos moschatus*), red deer, reindeer (*Rangifer tarandus*), sika deer (*Cervus nippon*) and wapiti (*Cervus canadensis*; Epe et al. 1997; Gibbons and Khalil 1988; Höglund et al. 2003). However, it is unclear whether this is indeed the correct host range, due to the problem of cryptic species, since only very limited consideration using molecular markers has been undertaken. Höglund et al. (2003) found that *D. eckerti* from red deer, moose, reindeer and musk ox group together in their molecular phylogenetic study, suggesting that it is a truly generalist species (at least amongst these hosts). We find that red deer is the primary host for *D. eckerti* within the region that we sampled. Although *D. eckerti* samples were recovered from all three deer species considered here, the vast majority of worms originated from red deer. However, to some extent, this may reflect sampling bias since 70 % of the deer sampled in this study were red deer. All of the six lungworms collected from fallow deer were identified as *D. eckerti*. The prevalence and abundance of dictyocaulid worms in fallow deer are very low in comparison to values in red deer (unpublished results). The distribution of fallow deer, which is patchy, may provide only a secondary host for the parasite, but more sampling is required to confirm this and to ascertain the focal host of *D. eckerti* and if this varies across its large range.

In contrast to the findings for *D. eckerti*, only 1 of 17 *D. capreolus* worms originated from red deer, with the rest sampled from roe deer. Thus, our data suggest that *D. capreolus* is a roe deer specialist in Hungary, although in Sweden it was also recovered from moose, suggesting a complex pattern of host associations (Höglund et al. 2003). To our knowledge, the occurence of *D. capreolus* in red deer (ID no. D22; Table 1) is a new host–parasite record. In addition, we believe this is the first time that *D. eckerti* has been recorded from roe deer confirmed by molecular analysis. As mentioned above, *D*. sp. S-HU isolates were collected exclusively from red deer. Thus, despite previous suggestions that *Dictyocaulus* species have a broad host spectrum (Eckert et al. 1992; Kassai 1999; Sprehn 1932), it is now clear from studies using molecular identification methods (including this one) that lungworm species generally infect more limited sets of hosts (Divina et al. 2002; Höglund et al. 2003).

Additional detailed genetic host–parasite data are required to clarify the extent to which all lungworm species use focal hosts across their range. Additionally, we demonstrate that *D. eckerti* and *D*. sp. S-HU share similar ecological habitats and the same host species (red deer). We could not identify any ecological factors underlying genetic differentiation between *D. eckerti* and *D*. sp. S-HU, and, hence, an interesting question is what factors exist to promote reproductive isolation between them. We did not investigate the closely related cattle lungworm, *D. viviparus*, here, but studies report that it is widespread in Hungary (Kassai and Holló 1962). As wild deer and cattle use often the same grazing sites, there could, in theory, be a high likelihood of cross-infection between deer and cattle lungworms. However, we did not observe any *D. viviparus* lungworms in deer. Earlier reports that deer species host *D. viviparus* in Hungary (Kutzer et al. 1987; Sugár 1990, 1994) may originate from erroneous identification based on morphology alone (Divina et al. 2000).

The levels of nucleotide diversity for *D. eckerti* samples are on a par with mtDNA diversity reported in various parasites of vertebrate hosts (∼2 % nucleotide diversity for mtDNA; Blouin et al. 1995, 1999; Blouin 2002; Braisher et al. 2004). *D. capreolus* samples showed lower genetic diversity than that for *D. eckerti* samples. Studies of *D. viviparus* in Sweden have indicated that mitochondrial sequences show moderate genetic diversity (Höglund et al. 2006; Hu et al. 2002). Our study indicates high nucleotide variation for wild lungworm species, with haplotype diversity approaching 1. It is striking that 70 haplotypes, belonging to three species, were identified from 103 lungworm specimens in Hungary. In comparison, for cattle lungworms in Sweden, 12 haplotypes from 252 cattle lungworm specimens were found (Hu et al. 2002). The *D. eckerti* populations included here are variable, but there were no clear differences between populations according to haplotype distributions. Our analyses detected higher levels of nucleotide variation in the *cox1* gene of lungworms from wild host species than were found in Sweden for cattle lungworms (Höglund et al. 2006). This comparison is not altogether straigthforward since the gene regions utilised in these studies are from two neighbouring fragments, as in this study we examined the 5′-end of *cox1* whilst in the cattle lungworm study the 3′-end of *cox1* was analysed. However, the mutation rate is only somewhat higher at the 5′-end of *cox1* than at the 3′-end in dictyocaulid lungworms (Gasser et al. 2012). Therefore, there appears to be considerably higher nucleotide diversity in *Dictyocaulus* lungworms from wild deer hosts than there is in those from farmed cattle hosts.

The estimated distributions of lungworm species examined in this study are larger than the sampling area. Whilst *D. capreolus* are recorded only from Europe (Spain: Carreno et al. 2009; Sweden: Divina et al. 2002; France: Durette-Desset et al. 1988) and Asia Minor (Turkey: Umur et al. 2012), *D. eckerti* is widely distributed in temperate regions worldwide, such as North America (Höglund et al. 2003), Europe (Epe et al. 1997), Siberia (Skrjabin et al. 1954) and New Zealand (Mason 1994; Gasser et al. 2012). Spatial structuring is evident where all populations of a species are not completely panmictic. The *Dictyocaulus* species considered here show three distinct population genetic classes across the examined range. First, *D. eckerti* has high host vagility and shows low population differentiation and consequently high *N*_*m*_ values. The high *N*_*m*_ values indicate that populations of *D. eckerti* show strong genetic connectivity. Second, *D. capreolus* in host populations with moderate vagility show moderate population structure, close to the critical *F*_ST_ = 0.2 value (Allendorf 1983). Genetic structure in *D. capreolus* appears distance-dependent, which may be a consequence of the limited dispersal behaviour of its roe deer hosts. Third, the *F*_ST_ of *D. viviparus* in hosts with very low vagility (i.e. cattle in farms) shows high population genetic structure far above the critical *F*_ST_ value. It is likely that *D. viviparus* has very low gene flow as the cattle hosts of *D. viviparus* are isolated by farms. Wild deer are not suitable hosts for *D. viviparus* (Höglund et al. 1999; Gasser et al. 2012). Thus, gene flow in *D. viviparus* populations is highly limited. Our results regarding the genetic structure of *D. capreolus* are similar to findings from a population genetic analysis of a different parasitic nematode of wild deer. Specifically, the white-tailed deer (*Odocoileus virginianus*) nematode parasite *Mazamastrongylus odocoilei* in North America shows high genetic diversity and moderate genetic structure (*N*_ST_ = 0.12 and 0.31; Blouin et al. 1995). However, it should be noted that *M. odocoilei* was studied using mitochondrial DNA sequences of the *ND4* region, which is more variable than the *cox1* locus, and also that the sites examined in America were situated at larger distances than those in our study.

Several studies have reported that the most important factor to impact on parasite population structure is the vagility of hosts (Levin and Parker 2013; McCoy et al. 2003). This may be especially true for trichostrongylid parasites, for which the infective larvae lack means of long-distance dispersal (Blouin et al. 1995). There are considerable differences in the dispersal patterns of the examined hosts. Fallow deer and red deer, which host *D. eckerti*, can migrate large distances, whilst roe deer migrate less and are considered to be a territorial species (Kropil et al. 2015; Cagnacci et al. 2011). Roe deer usually disperse individually (bucks) or in small goups (doe with fawn/s) during spring to autumn when lungworm infection is most likely. However, roe deer have two ecotypes in Hungary: forest-based roe deer live in groups of four to eight animals (SW-HU roe population), whilst field-based roe deer live in larger groups of dozens or even hundreds of individuals (E-HU roe population) during autumn to spring. The field-based bucks leave mixed sex groups in March, but females stay with the group until the second half of May, and fawns stay together for some additional weeks. We assume that cross-infection is more probable amongst group members (red deer and field-based roe deer) than it is amongst dispersed forest-based roe deer individuals. This hypothesis corresponds to observed levels of infection by *Dictyocaulus* in red and roe deer. The prevalence of infection values were 8.3, 13.0 and 46.6 % for forest-based roe deer, field-based roe deer and red deer, respectively (Sugár 1994, 1997). Prevalence is highest in the youngest age group of roe (33.3 %) and red deer (75 %; Sugár 1997). Therefore, the dispersal behaviour of hosts may be the best explanation for the different population genetic structures observed amongst *Dictyocaulus* species.

The differing levels of gene flow observed in lungworms have consequences for population dynamics and evolutionary potential (Barrett et al. 2008). Parasites such as *D. eckerti* with high gene flow between host species probably switch hosts often and may not experience such extreme population bottlenecks compared to worms restricted to a single host species. *D. capreolus* is reported to utilise an additional host species to roe deer in Sweden, the moose (Gibbons and Höglund 2002), which has a different dispersal behaviour. Consequently, one expectation is that populations of *D. capreolus* in Sweden may show lower genetic structure than those examined here in Hungary, particularly as the moose is a long-distance disperser (Sweanor and Sandegren 1989); it would be interesting to test this prediction.

Our results suggest that *D. eckerti* has not experienced a severe recent population bottleneck and that there was a population expansion ∼11,500 years ago (although these results should be interpreted with caution, e.g. see Morrison and Höglund 2005). Our estimate for a relatively recent *D. eckerti* population expansion is likely to be driven by the population expansion of its hosts. The population expansion time estimate is concordant with host migration and population expansion after the last Ice Age since climate warming began approximately 15,000 years ago (Denton et al. 2010). Further, archaeological and genetic data indicate that red deer and other wild ungulate hosts in Europe experienced population expansions approximately 10,000 years ago (Sommer et al. 2008). Red deer have three genetically differentiated populations in Europe: eastern, western and Mediterranean (Skog et al. 2009). Our sampling was performed on the eastern population of red deer, which arose from the Balkan glacial refugium. Sampling of *D. eckerti* across a larger spatial scale, including western and Medierreanean populations, may indicate greater genetic structure, following the main host’s genetic structure. In future studies, it would be interesting to examine whether lungworm genetic structure reflects that of its red deer hosts at larger scales across Europe and to what extent worms arising from different refugial populations have spread across distinct European host populations.

The high gene flow observed for *D. eckerti* in this study likely reflects a parasite population that extends over a larger spatial scale than our study area. When a population expands, it is expected to gain rare alleles, which we observe here for *D. eckerti*. The predicted large distribution, high genetic diversity and high gene flow for *D. eckerti* have important evolutionary consequences and offer the potential for new mutations to spread rapidly. The majority of red deer in Hungary are infected by lungworms during their first year (Sugár 1990, 1997), whilst only a low prevalence in roe deer was recorded (Sugár 1997), with the mean intensity of lungworm infection per individual higher in red deer than roe deer (Sugár, unpublished data). Taking into account lungworm distribution, host range, host vagility, prevalency and intensity, *D. capreolus* is likely to have a much smaller overall population size than *D. eckerti*. The high population size of *D. eckerti* could maintain high genetic diversity and an ability to respond quickly to forces of selection, and the impact of genetic drift should be negligible compared to that of natural selection. These predictions have considerable implications for lungworm management, particularly since high gene flow enhances the efficient evolution of resistance to treatment methods.
